# Impact of the war on forest ecosystem in Ukraine based on Sentinel-2 data

**DOI:** 10.1038/s41598-026-35744-7

**Published:** 2026-01-14

**Authors:** Adam Waśniewski, Alicja Rynkiewicz, Agata Hościło, Serhii Havryliuk, Oleh Chaskovskyy

**Affiliations:** 1 Department of Geoinformatics, Cartography and Remote Sensing, Faculty of Geography and Regional Studies, University of Warsaw, Warsaw, 00-927 Poland; 2 Centre of Applied Geomatics, Institute of Geodesy and Cartography, 02-679 Warsaw, Poland; 3 Present Address: National Centre for Emissions Management, Institute of Environmental Protection-National Research Institute, 02-170 Warsaw, Poland; 4 Department of Forest Inventory and Forest Management, Ukrainian National Forestry University, Lviv, 79057 Ukraine

**Keywords:** Forest ecosystem, Forest losses, Change detection, Environmental assessment, Sentinel-2, Ecology, Ecology, Environmental sciences, Environmental social sciences

## Abstract

Forests play a vital role in ecology, economy, urban planning, and social well-being, emphasising the importance of monitoring forest cover and its changes. The study evaluates the ecological impact of the military conflict on forest ecosystem in Ukraine using a time series of Sentinel-2 data and machine learning algorithms. Forest losses following the beginning of the war were derived using a change detection method across the study areas. Two types of forest loss were delineated: conversion of woody areas to non-woody cover, and burnt forest. Before the war, forest loss was predominantly due to the conversion of woody to non-woody cover, accounting for 74% of total changes, while forest fires represented the remaining 26%. Following the outbreak of the conflict, the total area of forest loss doubled. Notably, the proportion of forest converted to non-woody cover decreased to 66%, while the proportion of burnt forest increased to 34%, evidencing the severe impact of military operations on forest ecosystem. Of interest, the area of forest converted to non-wood cover doubled between, possibly reflecting an increased demand for wood due to the conflict, potentially driven by a rise in legal and illegal logging as a result of weakened governance and reduced enforcement of environmental regulations.

## Introduction

Forests play a crucial role in various aspects of human life, ranging from ecology and economy to urban development and social well-being. From an ecological perspective, trees are vital for conserving water, improving air quality, preserving soil, and providing oxygen to the atmosphere^1^. Economically, trees are essential for construction, transportation, and as a source of energy^2^. Urban trees, in particular, have a significant impact on the living standards of residents, enhancing air quality, reducing noise pollution, retaining rainwater, and regulating temperature and humidity levels, contributing to more sustainable urban development^3^. Given their multifaceted importance, continuous monitoring of forested areas is important for environmental preservation and mitigation of climate change.

Satellite remote sensing, originally initiated for military surveillance during the Cold War, has been essential in tracking environmental changes since the 1970s^4^. As Earth observation technology has developed, satellite imagery has become an indispensable data and methods for gathering quantitative information on forest monitoring. Remote sensing methods have the ability to monitor forests from space, where ground-based data is sparse or inaccessible due to geographical, political, or logistical reasons. This is especially critical for regions experiencing rapid environmental changes, exposed to natural disasters or prolonged military conflicts. Of interest, since the start of the Russian-Ukrainian conflict approximately 30% of the country’s territory was outside of the control of the Ukrainian government or contaminated with explosive objects^5^. Large areas have become unsafe and unsecured to conduct field inventories, collect reference data, or carry out field measurements^6,7^. This situation poses significant challenges for environmental monitoring and land management. Notably, an estimated 18% of protective plantations located in the eastern agroforestry region of Ukraine are now under threat due to ongoing hostilities^8^. Therefore, there is an urgent need to apply the remote sensing technologies, particularly satellite-based observation for assessing the status of the environment, estimating and monitoring the land cover changes in near real-time over the conflict areas. Cazzolla Gatti et al.^9^ has applied machine learning algorithm and Landsat 8 data to map forest losses in Ukraine, during the war. Landsat sensors provide data at 30 m spatial resolutions, which may not detect small damages, thus the use of for example Sentinel-2 data with 10 m spatial resolution can provide more detailed information on forest damages. The machine learning algorithms and optical Sentinel-2 data were successfully used to detect war-damage in agricultural fields^10^, whereas a synergy of Sentinel-2 and radar Sentinel-1 data was applied for the assessment of war-related building damage in Kyiv, Ukraine^11^.

Nowadays, the machine learning and deep learning algorithms applied to remote sensed data offer great effectiveness in the operational land cover classification and detection of land cover changes. Of interest, the machine learning algorithms such as Random Forest (RF), and Support Vector Machines (SVM) remain particularly popular due to their interpretability and lower computational cost compared to deep learning models^12^. RF is known for its stability and ability to handle high-dimensional, non-linear spatial data, making it a suitable choice for forest and land cover classification^13^ and have proven to be highly effective, particularly when applied to satellite imagery like Sentinel-2^14^. Several studies have demonstrated the superiority of RF over other machine learning models, such as support vector regression (SVR) and artificial neural networks (ANN), in mapping forest and detecting land cover changes^15^. Belenok et al.^16^ evaluated RF and SVM algorithms and Sentinel-2 data for mapping five land cover classes: water, forest, grassland, built-up, and other within the administrative boundaries of Kyiv. The authors stressed that RF consistently outperformed the SVM, achieving overall accuracies of 86% compared to 84% for SVM. Tokar et al.^17^ examined the performance of the RF in mapping the land cover in the Prykarpattya region of Ukraine using Sentinel-2 and Landsat-8 imagery and concluded that forest classes achieved moderate overall accuracy of 69–74%, although as the delineation of deciduous and coniferous forest, presented challenges with user and producer accuracies in the range of 60–70%. A study by Hościło and Lewandowska^18^ confirmed the potential of multi-temporal Sentinel-2 data for precise delineation of forest cover, forest types, and tree species at a regional scale over the part of the Carpathian Mountains as well as demonstrated the reliability and effectiveness of RF, for large-scale forest monitoring. The RF and Sentinel-2 data have been also successfully applied to map forest cover in other part of the World, e.g., Gabon^19^ and China^20^.

Comparing forest classification results over time is a commonly used method for detecting changes in forest cover. However, this approach is prone to errors arising from inaccuracies in the input data, which can affect the reliability of change detection results^21^. As an alternative, direct change detection methods focusing on analysis of the multi-temporal data without relying on prior classification, offer improved accuracy^22^. For example, the approach by Hansen et al.^23^, which utilizes multi-year Landsat data to detect forest cover changes, is particularly effective for retrospective analysis. More recent innovations, such as the near real-time (NRT) forest monitoring system developed by Pacheco-Pascagaza et al.^21^ using Sentinel-2 data, have further advanced forest monitoring capabilities. This system achieved an overall accuracy (OA) of 93% in detecting forest losses in Mexico and Colombia. However, its reliance on local training data, which may not always be available or accessible, especially in conflict zones or regions experiencing rapid environmental changes, where such data may be difficult to obtain.

A promising alternative is a two-phase approach that combines rapid analysis of spectral indices to facilitate the collection of training samples with a RF classifier. This method has been successfully applied to detect vegetation losses at regional scale in Poland and Norway^24^. The algorithm was applied to Sentinel-2 data from 2018 to 2021 reaching an OA of 97% or higher across all time intervals and both study areas.

This study aims to support forest monitoring in areas affected by military conflict by deriving reliable and quantitative data on forest changes using artificial intelligence and Earth observation data. In the first step, the RF machine learning algorithm was applied to Sentinel-2 data to map forest extent and dominant leaf types (coniferous and broadleaved) for the reference year 2020, prior to the onset of the armed conflict in Ukraine. Next, forest loss within the identified forest mask was detected for two-time intervals: 2020–2021 (pre-conflict) and 2021–2022 (post-conflict). The analysis focused on Ukraine’s most forested regions: Lviv, Kyiv, Zhytomyr and Kharkiv. To evaluate the accuracy and relevance of the results, the derived forest loss data were compared with forest loss provided by the Global Forest Change (GFC) dataset, which is based on Landsat data and follows the methodology developed by Hansen et al.^23^.

## Materials and methods

### Study area

**Fig. 1:**
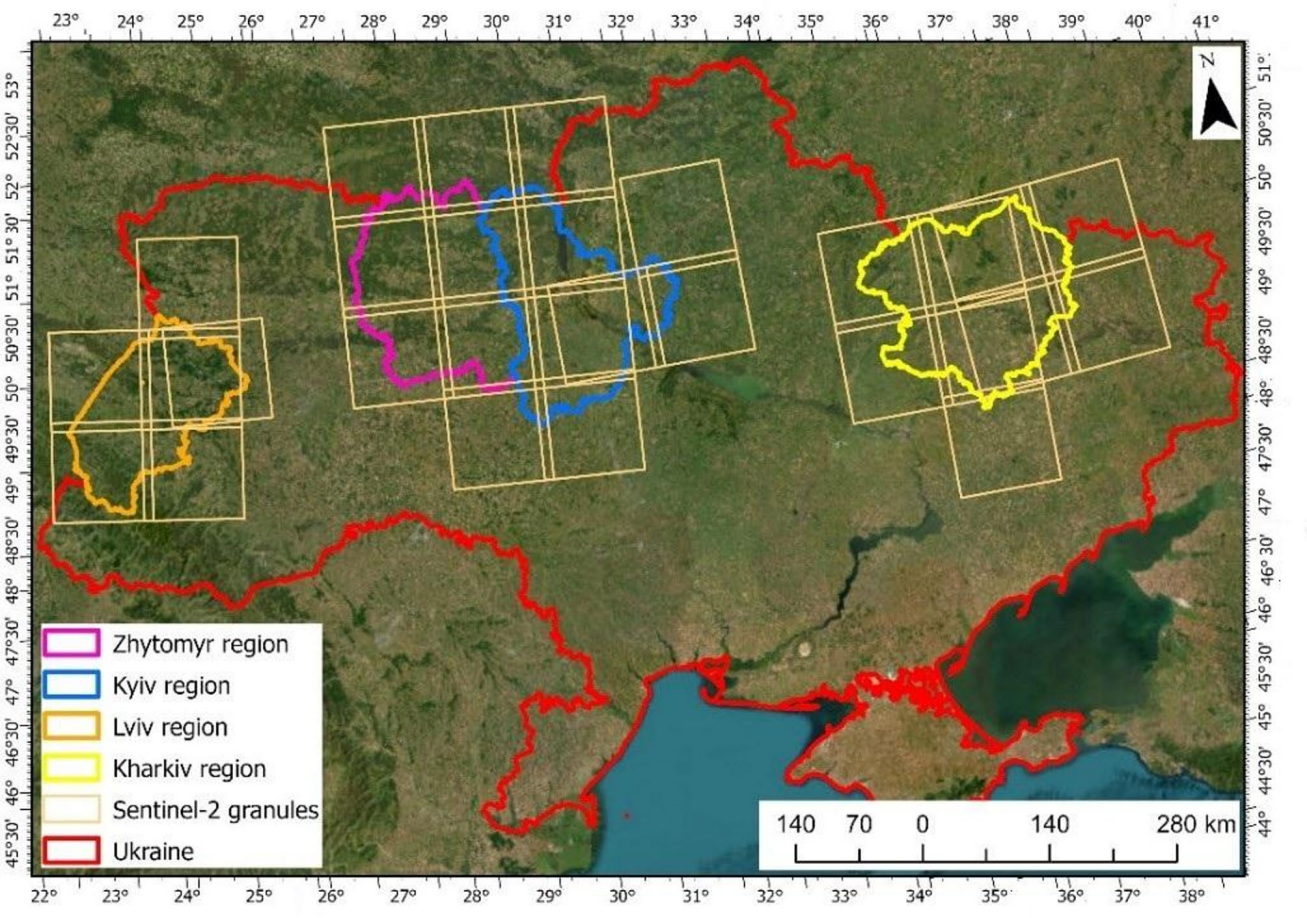
Study areas with the overlapped extent of the Sentinel-2 granules (basemap: World Imagery, ArcGIS Pro; the map image is the intellectual property of Esri and its licensors, used under license, 2025. All rights reserved).

The study was performed over four regions of Ukraine—Lviv, Kyiv, Zhytomyr and Kharkiv (Fig. 1). The Lviv region is situated on the south-west border of Eastern European plain and the west part of the north mega slope of Ukrainian Carpathians, on the border with Poland. The landscape of this region is highly varied. In the southern part, the mountainous terrain is covered predominantly by secondary broadleaf and coniferous forest. The lowlands of the central part are mostly covered by arable land with woody patches, whereas the northern part of the region is densely forested. Both deciduous and coniferous trees are present.

The Kyiv, Kharkiv, and Zhytomyr regions are situated in the central northern part of Ukraine, along the border with Russia, within the Dnipro River basin. All regions were heavily affected by severe Russian military operations starting in February 2022. The Zhytomyr region is densely forested, with the very southern part dominated by arable land. In the Kyiv region, forests are mainly located in the northern part, whereas the eastern and southern areas are primarily agricultural. The landscape of both regions is relatively flat. The Kharkiv region, in contrast, has a limited forest cover, with extensive agricultural areas dominating the landscape, and it was among the areas most exposed to intensive military activities during the initial phase of the armed conflict. The landscape of these two regions is rather flat. According to the national statistics, the area of these four regions is around 112 047 km^2^^25^.

### Data

#### Sentinel-2 satellite images

Sentinel-2 A and 2B images were downloaded from the Creodias EO data platform. The cloud free Sentinel-2 images captured within the vegetation season, between April and September 2020 were selected for further analysis. In the absence of cloudless images in 2020, we use the images acquired +/−1 year. For each Sentinel-2 granule, 3 to 5 images were chosen. In total, 123 Sentinel-2 images were used in the classification of forest and dominant leaf type. The images were atmospherically corrected (Level-2 A), with bottom-of-atmosphere reflectance calibration, the radiometric adjustment was not performed. In total 10 spectral bands of Sentinel-2 were used in this study: four bands at 10 m spatial resolution (B2 Blue, B3 Green, B4 Red, B8 NIR) and six bands at 20 m spatial resolution (red edge bands—B5, B6, B7, B8A and B11 SWIR1, B12 SWIR2), the 60 m spatial resolution bands were excluded from the analysis. The 20 m resolution bands were resampled to 10 m using a bilinear resampling method to ensure spatial consistency.The classification of forest range and dominant leaf type was conducted on the stacked images.

Detection of forest losses was performed using a cloud-based Google Earth Engine (GEE) platform, which provides access to a time series of Sentinel-2 images. The Sentinel-2 Level-2 A data (COPERNICUS_S2_SR) containing orthorectified atmospherically corrected surface reflectance and Sentinel-2 Cloud Probability product (COPERNICUS _S2_CP) for cloud masking have been used. The SCL band provided with the Sentinel-2 Level-2 A product was used to mask cloud shadows.

#### Reference dataset

Due to a lack of accurate national reference data on land cover or forest extent over the study area, we used the existing global land cover maps: ESA World Cover 2020^26^ and Copernicus Global Land Cover Map (CGLS-LC100) Collection 3 for 2019^27^.

The ESA World Cover is an open global land cover product available at 10 m spatial resolution. It was generated using a classification of radar Sentinel-1 and optical Sentinel-2 data for the reference year 2020. In total, 11 land cover classes: forest, shrubland, grassland, cropland, built-up, bare/sparse vegetation, snow and ice, permanent water bodies, herbaceous wetland, mangroves, moss and lichen were mapped with the overall accuracy of 74.4%^26^.

The Copernicus Global Land Cover Map (CGLS-LC100) was developed annually for the time frame of 2015–2019, at 100 m spatial resolution based on satellite data provided by PROBA-V and Sentinel-2. The CGLCM contains three land cover levels: (1) single class forest; (2) open/closed forest and (3) all forest types. In the first classification level called *single class forest* the following land cover classes are featured: forest, shrubland, herbaceous vegetation, herbaceous wetlands, moss and lichen, bare and sparse vegetation, cropland, built-up, snow and ice, permanent water bodies. At the second level, the forest is divided into: closed and open forest. Then, at the lower classification level (all forest types) open and closed forest are divided into six sub-classes: evergreen needle-leaved, deciduous needle-leaved, evergreen broadleaved, deciduous broadleaved, mixed type and unknown type.

### Methods

**Fig. 2:**
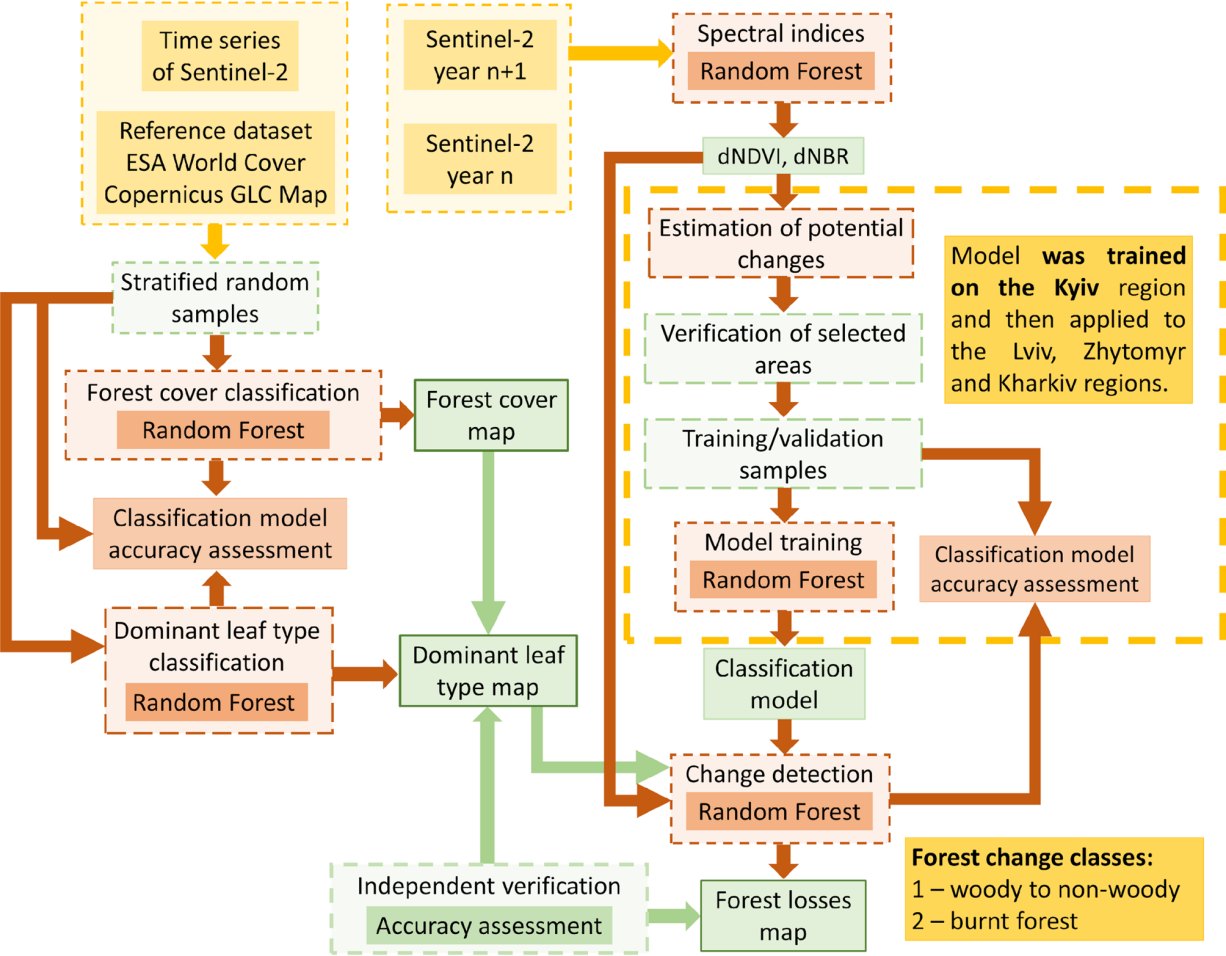
Scheme of applied methods for classification and change detection analysis.

The methods applied in this study consisted of a series of analyses and data processing steps. The workflow diagram illustrates the input data, preparation of reference samples, forest cover and dominant leaf type classification, followed by change detection, which included the calculation of indices, estimation and classification of changes, and independent validation of the results. The methodological workflow with its subsequent stages is presented in Fig. 2.

#### Preparation of stratified reference samples for the classification

Reference samples in a form of point were created randomly over the study areas. For each point, the information on land cover classes: coniferous trees, broadleaved trees, built-up, grassland, cropland, wetland, bare soil and water was extracted from the CGLS-LC100 and ESA World Cover. The samples were prepared individually for each Sentinel-2 granule according to the following rules: (1) a 10 m internal buffer was applied to the reference datasets to avoid placing points near the edges of land cover classes; (2) a minimum distance of 20 m was maintained between sampling points; and (3) 2 points per km^2^. The number of points for each land cover class was set by trial-and-error method to optimize processing time and to provide sufficient spectral reflectance information. Number of reference samples depends on the area and heterogeneity of each land cover class. The following three attributes were assigned for each reference sample: (1) land cover class, (2) forest or non-forest, and (3) coniferous or broadleaved trees. Finally, the reference samples were verified automatically using the analysis of the spectral features according to the methodology developed by Waśniewski et al.^28^. Table 1 presents the total number of reference samples for each type of land cover used for the classification.

**Table 1:** Total number of reference samples per land cover class for all classified Sentinel-2 granules.

Land cover class		Number of reference samples
Forest	Coniferous trees	63 090
	Broadleaved trees	56 190
Non-forest	Sealed surfaces	17 231
	Grasslands	26 703
	Croplands	156 030
	Bare soil	11 672
	Wetlands	10 967
	Water bodies	19 611
	Total	361 494

#### Classification of forest cover and dominant leaf type

The classifications of forest cover and dominant leaf type were performed for the reference year 2020, individually for each Sentinel-2 granule using the Random Forest (RF) classifier^13^. An exception was made for the Kharkiv region, where, due to the small forest areas, a single model was trained on one granule and then applied to the remaining granules.

The classification process was conducted in two steps: (1) classification of the forest cover, and (2) classification of the dominant leaf type (coniferous or broadleaved) within the forest mask obtained from the first step. The classifications were implemented in the Python environment on cloud computing virtual machines, using mainly the scikit-learn Python library. The RF classification model was trained using 60% of randomly selected reference samples for each class, while the remaining 40% of samples were used to assess the classification model’s accuracy.

#### Change detection

The forest changes were detected within the forest mask for 2020 for two-time intervals: 2020–2021 and 2021–2022. It was implemented in the GEE platform that provides access to a repository of satellite images and powerful cloud computing facilities for spatial analysis^29^. The change detection method was based on the direct classification of changes, following the approach developed by Rynkiewicz et al.^24^. The changes were detected using annual seasonal mosaics of Sentinel-2 images, created through temporal aggregation of the median spectral reflectance values during the vegetation season (June to August), as proposed by Carrasco et al.^30^. These temporal mosaics were generated for each study area for the years 2020, 2021, and 2022. The following three classes were distinguished: 1—no change, 2—woody areas converted to non-woody cover (e.g. clear cuts, logging, forest damage), and 3—burnt forest.

In the first step, the vegetation index differencing^31^ was calculated to indicate the potential changes over the Kiev region. Two common spectral indices, the Normalized Difference Vegetation Index (NDVI) and the Normalised Burn Ratio (NBR), were calculated for each annual mosaic. Both indices are associated with important biophysical parameters and are sensitive to forest regeneration and disturbances^32^. Next, the differences, dNDVI and dNBR, were calculated from the annual NDVI and NBR values for two consecutive years. A threshold of greater than three standard deviations in the dNDVI and dNBR values was applied to identify potential forest loss areas. Subsequently, nine reference areas covering change and no change classes were selected based on manual verification supported by the Sentinel-2 mosaics and Google Maps. The size of the reference areas ranged from 4 to 400 hectares. A stratified random sampling method was applied to generate reference points within the selected areas for each class. The points were then split into training and validation datasets using 7:3 ratio. The training samples were used to parametrise the Random Forest (RF) classifier and to derive the change detection product for the period 2020–2021 over the Kiev region. The classification was carried out using a data stack comprising annual Sentinel-2 mosaics for two consecutive years, along with dNBR and dNDVI values. The RF classifier with 100 trees was employed. In the next step, the pre-trained classification model was applied (using a transfer learning approach) to the other study regions for the periods 2020–2021 and 2021–2022. Finally, changes with an area less than or equal to 400 m^2^ (equivalent to 4 pixels) were considered unreliable and excluded from the results.

### Accuracy assessment

The accuracy assessment of the forest cover and dominant leaf types classification models was conducted individually for each Sentinel-2 granule using the verification samples. For the change detection classification model, the accuracy was calculated using the validation samples from the Kyiv region for the period 2020–2021. The following accuracy indicators were calculated: overall accuracy (OA), Kappa coefficient and F1 score, which is represented as the weight average of user’s (UA), and producer’s accuracy (PA).

Additionally, independent verification of the final maps of forest cover, dominant leaf type and forest changes was conducted through visually assessment using the Sentinel-2 mosaics and images available on the Google Earth platform. A stratified random sampling method was applied to generate verification samples for each class and time interval. To minimise edge effect, the extent of each of forest classes was shrunk by 10 m inward. Verification samples were converted into polygons of 10 by 10 m, and a total of 100 polygons per were visually verified. Finally, an error matrix was constructed and UA, PA and OA were calculated for each study region and each map.

## Results

### Forest cover and dominant leaf types

The areas of forest cover derived in this research, together with dominant leaf types and forest changes in each study region, are presented in Table 2 The Zhytomyr region has the largest forest area, covering 1,212,749 ha, which accounts for 40.6% of the region’s total area. The Lviv and Kyiv regions have comparable forest areas, with approximately 807,144 ha (37.0% of the region) and 812,360 ha (28.1%), respectively. In contrast, the Kharkiv region has roughly half as much forest, with only 431,179 ha, representing 13.7% of its area. Broadleaved trees are more prevalent in the Lviv (69.0%) and Zhytomyr (52.3%) regions compared to coniferous species. In contrast, the Kyiv region has a higher proportion of coniferous trees, which comprise 55.5% of the total forest area.

**Table 2:** Area of forest cover, dominant leaf type and forest loss in the study regions. In brackets, the percentage of forest area within the region and the percentage of the dominant leaf type within the total forest area.

	Area in hectares [%]				Forest loss 2020–2021 [ha]		Forest loss 2021–2022 [ha]	
Region	Region area	Forest cover	Coniferous	Broadleaved	Woody to non-woody	Burnt forest	Woody to non-woody	Burnt forest
Lviv	2 182 326	807 144 [37.0]	249 946 [31.0]	557 198 [69.0]	1 964	51	4 370	127
Kyiv	2 894 914	812 360 [28.1]	450 456 [55.5]	361 904 [44.5]	2 666	2 415	5 659	5 991
Zhytomyr	2 983 789	1 212 749 [40.6]	578 562 [47.7]	634 187 [52.3]	5 806	1 186	10 622	1 456
Kharkiv	3 143 488	431 179 [13.7]	–	–	1 518	655	2 543	4 632

### Analysis of forest changes

**Fig. 3:**
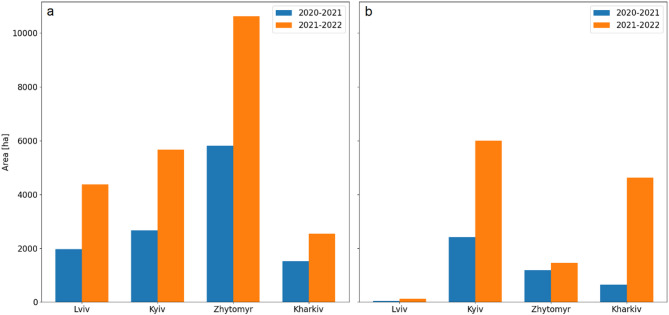
The area of forest losses: (**a**) woody to non-woody cover, and (**b**) burnt forest within four study regions for the periods 2020–2021 and 2021–2022.

The total area of forest loss within the four regions for the period 2020–2022 was equal to 51,661 ha, and increased from 16,261 ha in 2021 to 35,400 ha in 2022, following the start of the armed conflict (Fig. 3).

Before the war, the forest losses were predominantly associated with the conversion of woody to non-woody cover, accounting for 74% of all changes (11,954 ha), while the remaining 26% (4,307 ha) resulted from forest fires. After the onset of the armed conflict, the total area of forest loss doubled. The proportion of forest converted to non-woody cover decreased to 66% (23,194 ha), while the area affected by fire increased to 34% (12,206 ha), demonstrating the severe impact of military operations on forest ecosystems. The greatest forest damage was recorded in the Kyiv region, where 11,650 ha of forest was lost, representing 1.4% of the region’s total forest area, with more than half of this loss caused by fire. In the Zhytomyr, total forest loss was slightly higher at 12,078 ha; however, 88% of the loss was owing to the conversion to non-woody cover. The highest proportion of total forest area lost in 2022 (1.5%) was observed in the Kharkiv region, where 7,175 ha were affected, of which 35% was burnt. For comparison, in 2021, only 0.5% (655 ha) of forest loss in the Kharkiv region was caused by fires. Of interest, in the Lviv region, the proportion of forest converted to non-wood cover remained consistent across both periods (97%), although the total area of forest loss doubled (Table 1).

**Fig. 4:**
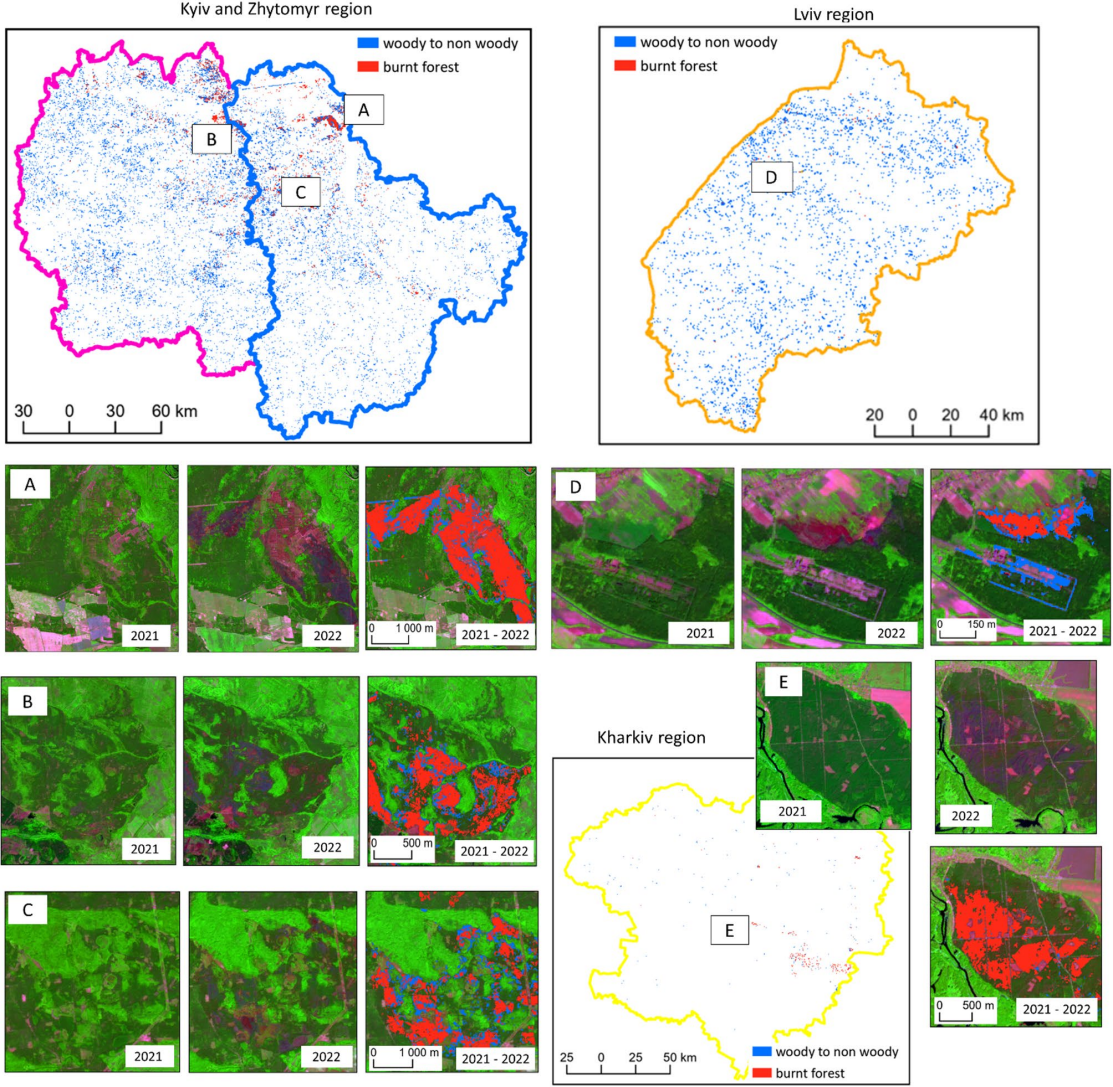
Forest losses in the study regions between 2021–2022 (**A**, **C**) Kyiv; (**B**) Zhytomyr, (**D**) Lviv, and (**E**) Kharkiv, basemap: Sentinel-2 composite: bands 12, 8, and 4).

Figure 4 illustrates the spatial distribution of forest changes in Ukraine between 2020 and 2022, focusing on regions significantly affected by war-related disturbances. The figure presents forest cover changes in the Kyiv, Zhytomyr, Lviv, and Kharkiv regions, highlighting areas classified as woody to non-woody transitions (in blue) and burned forest (in red). The main maps show the overall spatial extent of these changes, while insets (A–E) provide detailed views of specific forest loss areas, based on Sentinel-2 imagery from 2021 to 2022.

### Accuracy assessment and independent verification of the products

Overall, the classification accuracy for both forest cover and dominant leaf type is high, with slight variations across different granules. The overall accuracy (OA) of the forest cover and dominant leaf type classification models for three regions reached above 95%. In the Kyiv and Zhytomyr regions, the OA of forest cover ranged from 97 to 98%, the Kappa values 94–97%, and the F1 score 97–99%. The highest accuracies were observed in the central and southern parts of the study area, whereas the lowest in the northern and north-western part. In the Kharkiv region, the OA, Kappa and F1 were relatively high 98%, 96% and 97%, respectively.

The accuracy of the dominant leaf type classification model was slightly lower compared to the forest cover classification. The highest and most stable model accuracy was observed in the Kyiv region, with the OA ranging from 95% to 98%, In contrast, the Zhytomyr region generally exhibited OA values 1–2% points lower, with the best results observed in the southern granules and the lowest in the northern ones. For the change detection model, the OA in the Kyiv region reached 98%, with the Kappa coefficient of 97%. However, the forest fire areas were generally underestimated, with the producer’s accuracy (PA) of 94%, as 25 verification samples of burnt forest were misclassified as woody to non-woody transitions.

The results of the independent verification of the final products: forest cover, dominant leaf type and forest changes confirmed a high level of accuracy (Table 3). OA exceeded 96% for forest cover and dominant leaf type, and ranged 83 and 89% for forest change detection. Forest cover was mapped with a high UA of 98% and a PA of 94%, indicating low commission and omission errors, respectively. Similarly, the classification of dominant leaf types achieved an OA above 97% and a Kappa coefficient of 94%, reflecting excellent reliability. Broadleaved forest was identified with particularly high accuracy (UA of 97% and PA of 96%), while coniferous forest showed slightly lower, but still robust accuracy results. The accuracy of the forest fires detection was consistent across time periods, with UA exceeding 70% and PA over 90%. Misclassification was mostly common at the edge of the burned areas, which were often labelled as woody to non-woody transition. Additionally, shadows at forest boundaries were occasionally misclassified as burned areas. The woody to non-woody conversion was detected with a UA of 94% in 2021 and 79% in 2022, while the PA for this class remained above 76% in both years.

**Table 3:** Independent verification of the forest cover, dominant leaf type and forest changes [%].

		Land cover class	User’s accuracy	Producer’s accuracy	Overall accuracy	Kappa coefficient	
Tree cover		Forest	98	94	96	92	
		Non-forest	94	98			
Dominant leaf type		Broadleaved	97	96	97	94	
		Coniferous	96	97			
Forest loss 2020–2021		No change	99	86	89	84	
		Woody to non-woody	73	79			
		Burnt forest	73	99			
Forest loss 2021–2022		No change	100	77	83	74	
		Woody to non-woody	79	76			
		Burnt forest	70	90			

## Discussion

In this study, we addressed the urgent need to assess and monitor the degradation of forest ecosystems in Ukraine caused by the ongoing military conflict. A recent study by Filho et al.^33^ highlighted a negative impact of the war on the environment, including land degradation, air and water pollution, damage to wildlife and habitats, agricultural land, forest ecosystems and protected areas with soil degradation being a notable concern. To date, there is still a limited number of studies examining the impact of the military conflict on Ukraine’s natural resources. In our research, we provided evidence of the war’s impact on forest ecosystems in selected regions of Ukraine using the RF machine learning algorithm and Sentinel-2 data. We classified forest cover and dominant leaf type separately for each Sentinel-2 granule, achieving high overall accuracy—over 95% for forest cover and 94% for dominant leaf type. Additionally, these results were confirmed through independent validation, which showed accuracies exceeding 96% for both products and over 83% for forest change detection (Table 3). Our findings are in line with, or exceed the classification accuracies reported in other Sentinel-2-based studies^17,18,34^. Although the reference samples used for training were initially derived from the global land cover products: CGLS-LC100 and ESA WorldCover, with reported overall accuracies of approximately 75–80%, their quality was substantially improved through an additional verification process following the methodology proposed by Waśniewski et al.^28^. In particular, all reference samples were cross-checked using Sentinel-2 spectral reflectance values to ensure that the assigned labels accurately represented the actual land cover classes. This verification approach effectively reduced uncertainties in the global land cover maps used for the preparation of training data and enabled the Random Forest classifier to achieve higher overall accuracy than the original reference products. It should also be noted that, due to the ongoing military conflict in the study area, it was not possible to conduct an in-situ field campaign. Therefore, the use of globally available land cover products subject to additional automatic verification ensures reliable and consistent source of reference data for classification of forest area. It needs to be highlighted that using global land-cover products without additional quality checks and verification can lead to misclassification, reducing the accuracy of the final products, particularly in areas affected by severe conflict resulting in the significant changes in the land cover.

The RF was applied to delineate forest cover and dominant leaf type for the year 2020. Within the resulting forest mask, forest losses were then detected for the periods 2020–2021 and 2021–2022 across four regions: Kyiv, Zhytomyr, Kharkiv, and Lviv. Before the war, forest losses were predominantly associated with the conversion of woody to non-woody cover, accounting for 74% of the total changes, while the remaining 26% resulted from forest fires. Since the beginning of the armed conflict, the total area of forest loss doubled. The proportion of forest converted to non-woody cover decreased to 66%, while the area affected by fire increased to 34%, highlighting the severe impact of military operations on forest ecosystems. In 2022, the Kharkiv region recorded the highest proportion of forest loss, with 35% of its losses attributed to deforestation, followed by the Kyiv and Zhytomyr regions.

**Fig. 5:**
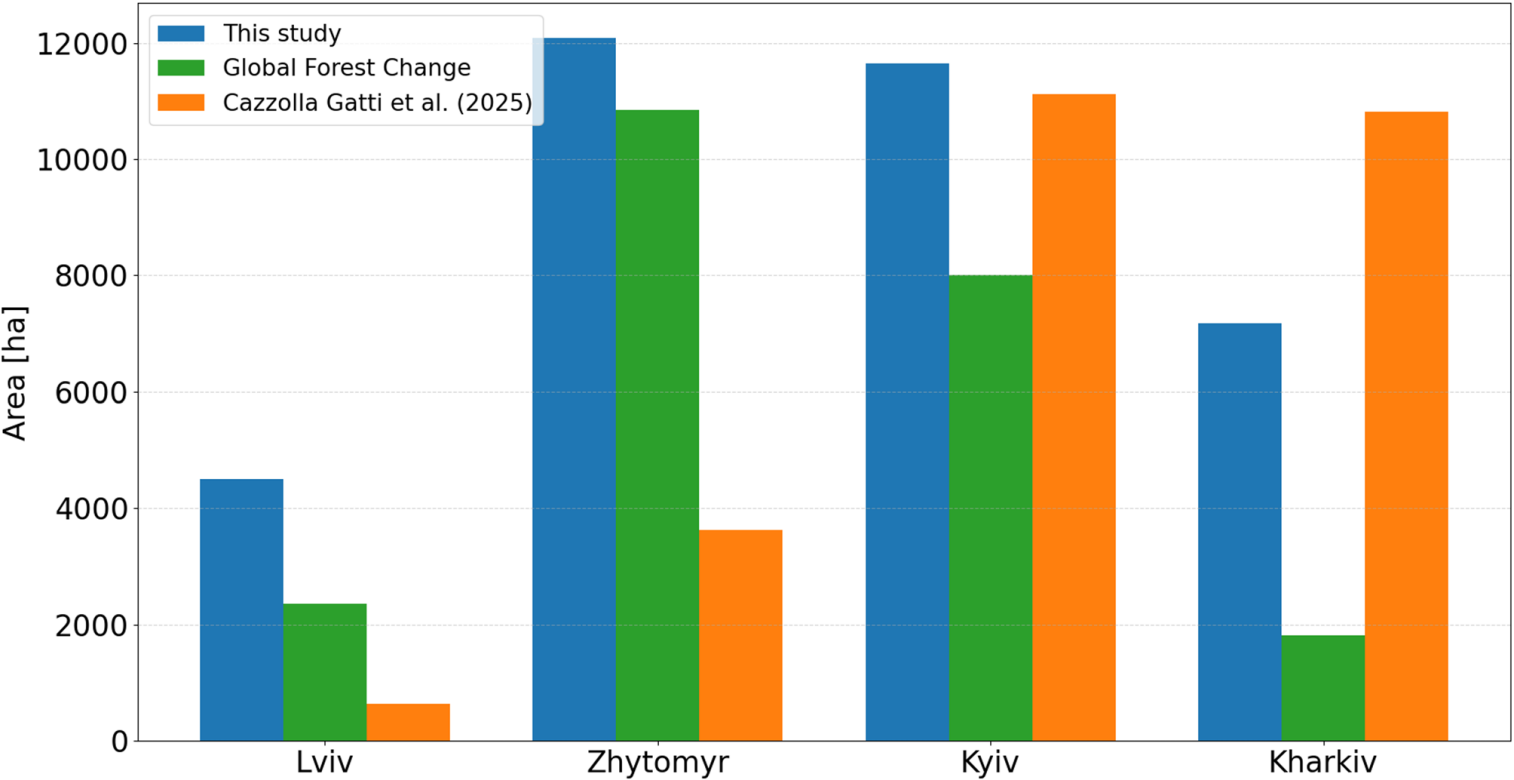
Area of forest losses in hectares in 2022 derived by our study, the Global Forest Change, and Cazzolla Gatti et al.^9^.

Of interest, the area of forest loss derived in our study differs from those recently published recently by Cazzolla Gatti et al.^9^ and provided by the Global Forest Change (GFC), based on the methodology proposed by Hansen et al.^23^ (Fig. 5). While Cazzolla Gatti et al.^9^ and GFC rely on Landsat data with 30 m spatial resolution, our analysis was based on Sentinel-2 data with higher spatial resolution. Notably, there is a significant discrepancy between the results reported by Cazzolla Gatti et al.^9^ and those of GFC in the study regions, despite the fact thatCazzolla Gatti et al.^9^ used the GFC dataset to train their RF model.

In our opinion, the discrepancy is primarily caused by the methodology applied by Cazzolla Gatti et al.^9^. The authors trained the forest loss classification models using data from only two small study areas and then transferred the pre-trained model to the rest of the country. In addition, they acknowledged that their results might slightly underestimate forest losses. On the other hand, they noted that riparian vegetation was sometimes misclassified as forest loss. Unfortunately, the authors do not provide detailed information on the accuracy of the RF models. They also stated that no clear-cuts have been detected, and all identified losses were related to fires. This claim is questionable, as the reference data used to train the RF models (Global Forest Change dataset) contains only a single class of forest loss class and does not distinguish between different causes of forest losses. Moreover, the area of forest losses reported by Cazzolla Gatti et al.^9^ is much higher than those we identified in the burnt forest class in the most fire affected regions - Kharkiv and Kyiv as well as those reported by the GFC under the general forest loss category.

In general, we reported higher values of forest loss for 2022 compared to GFC across all four regions. The largest differences were observed in regions severely affected by the war, such as Kharkiv and Kyiv. The underestimation of the forest loss in the GFC dataset may be attributed to the coarser spatial resolution of Landsat data (30 by 30 m) in contrast to the higher resolution of Sentinel-2 data (10 by 10 m) used in our study.

**Fig. 6:**
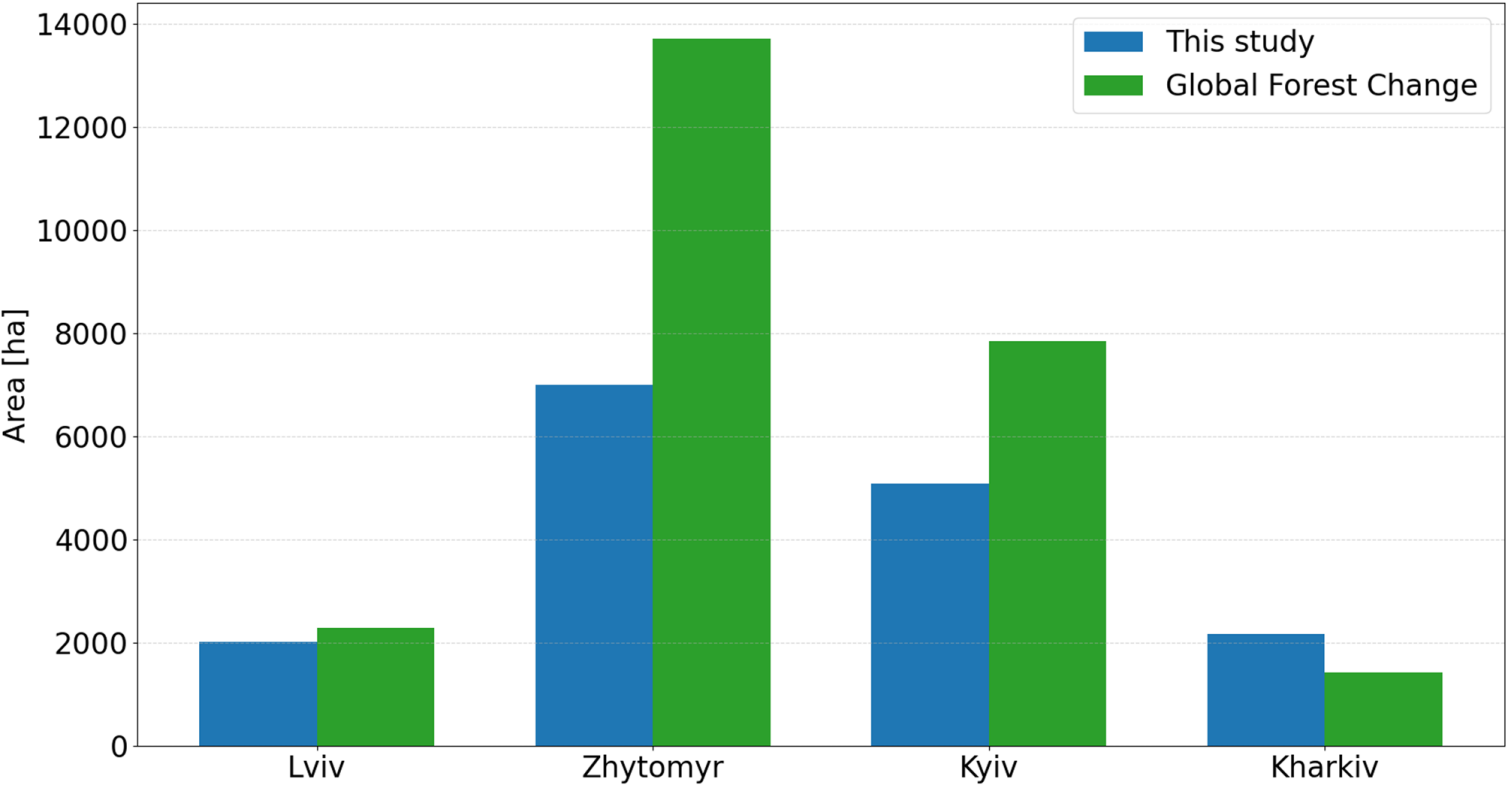
Area of forest losses in hectares in 2021 derived by our study and in the Global Forest Change.

**Fig. 7:**
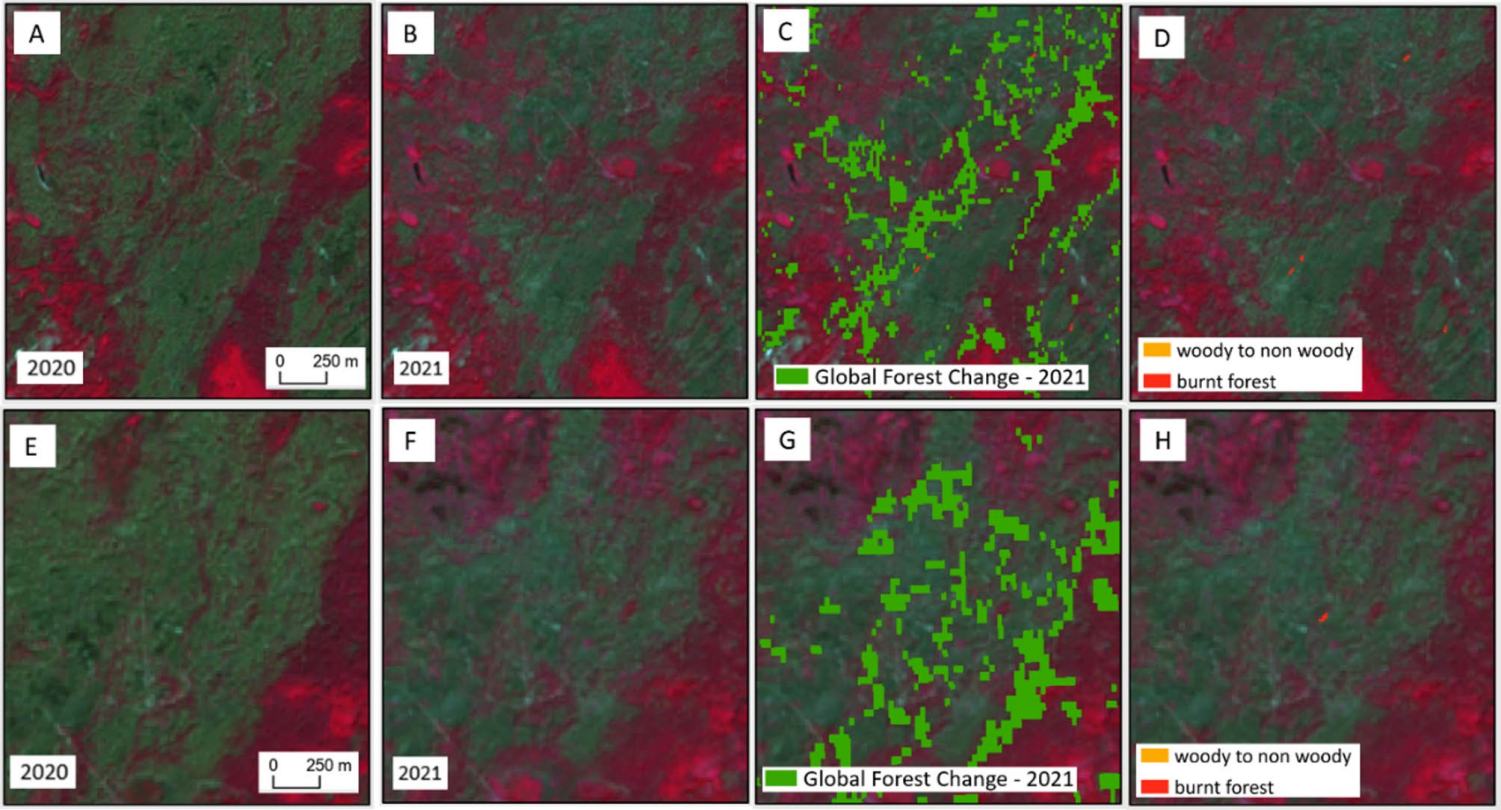
Overestimation of forest loss in the Global Forest Change (GFC) for 2021 in the Kyiv region. (**a**, **e**)—Sentinel-2 composite (bands 8, 4, 3) from 2020; (**b**, **f**)—Sentinel-2 composite (band 8, 4, 3) from 2021; (**c**, **g**)—GFC tree cover loss extent for 2021 overlaid on Sentinel-2 imagery; (**d**, **h**)—forest losses detected in this study.

Interestingly, a comparison of forest losses in 2021, priory to the war, revealed that our study reported slightly lower values in three regions compared to those reported by the GFC (Fig. 6). The GFC product appears to overestimate forest loss, particularly in areas previously affected by fires and in case where regenerated forest patches were misclassified as loss. Similar findings were reported by Galiatsatos et al.^35^ in their assessment of GFC datasets for national forest monitoring and reporting. They emphasized that the automatic classification algorithm used in the GFC product may have difficulty to accurately delineate change boundaries, leading to overestimations in the final tree cover loss map. Figure 7 presents examples of areas where forest cover loss was overestimated in the GFC dataset, while no such loss was detected in our study.

In the methodological approach, forest loss was detected using a RF classifier initially trained on data from the Kiev region. This pre-trained model was subsequently applied to the three additional regions over multiple time periods. The independent validation demonstrated that the model exhibited good transferability across regions and over time. However, some misclassification errors were observed in the Lviv region, likely owing to the relatively low number of fires and the presence of small burn scars, which are more difficult to detect accurately. It is important to emphasise that the effectiveness of the model transferability depends on similarity of landscape characteristics and the geographical proximity of the target regions to the area used for model training. Matsala et al.^36^ investigated how the war influences forest fire risks and highlighted the importance of distinguishing.

clear cuts from other disturbances such as burned areas, windthrows, and insect outbreaks. They applied the Tasseled Cap Transformation to differentiate clear-cuts from other disturbances and achieved an overall accuracy of 95%. The authors also stressed the necessity of distinguishing between fires affecting coniferous and deciduous forests, potentially down to species level, due to the variation in fire susceptibility among tree species. Their analysis focused only on fires in coniferous forests. Our research addresses the gaps identified by Matsala et al.^36^, particularly regarding fire-affected areas both dominant leaf types and the delineation of forest areas converted to non-wood cover. Nonetheless, future research should aim to further disaggregate this class into clear cuts and degraded or logged forests.

Based on our results, coniferous forests, being more susceptible to fire, accounted for a significant proportion of losses, particularly in the Kharkiv, Kyiv and Zhytomyr regions, which experienced severe military-related disturbances. The higher fire sensitivity of coniferous forests, especially pure pine stands, increases the probability of forest losses in these areas. In contrast, the Lviv region, dominated by mixed and broadleaved forests, showed relatively lower levels of fire related forest losses, likely because of the lower vulnerability of these forest types to fire and reduced war activity. The observed trends in forest loss highlight the complex interaction between forest composition and ongoing conflict, underlining the need for region specific approaches to forest management and monitoring during and after periods of military activities.

In the analysed regions, the area of forest converted to non-wood land cover doubled between the two periods, reaching its highest levels in the Zhytomyr, followed by Kyiv, Lviv and Kharkiv regions. This trend may indicate a growing demand for wood following the start of military aggression, including an increase of clear cutting and illegal logging driven probably by weakened governance and reduced enforcement of environmental regulations. Infrastructure development for military purposes, such as the construction of military bases, fortifications, and roads, often requires clearing forested areas. Moreover, the displacement of local populations leading to unmanaged or abandoned forests that may become degraded or repurposed for agriculture or other land uses. Increased pressure on natural resources from military operations may contribute to unsustainable exploitation of forests. Economic instability and inadequate forest management practices have further accelerated unregulated deforestation and land-use changes. Additionally, many areas affected by the war remain dangerous due to the presence of unexploded ordnance, making it impossible to manage them in a typical manner.

In addition, the CO₂ emissions from military activities, soil degradation, water pollution, overexploitation of limited resources, and widespread loss of forests and green spaces are causing profound environmental impacts in Ukraine, with recovery potentially taking decades^33^. The decline in forest ecosystems threatens biodiversity, which beyond its intrinsic value, provides important ecosystem functions essential for human well-being^37^. In a country like Ukraine, where forest resources are already scarce and overexploited, ecosystem services such as pollution control, water filtration, primary production, soil formation, climate regulation through carbon sequestration, agroecosystem services, and recreational opportunities are invaluable^38^. The observations underscore the urgent need for robust up-to-date land monitoring system during the war and post-war environmental policies in Ukraine. Prioritizing forest restoration, reforestation, and protection is essential to prevent further biodiversity loss and mitigate long-term ecological damage^39^. Policy frameworks should also address the drivers of deforestation beyond war-related destruction, such as illegal logging and unsustainable land-use changes. Furthermore, public awareness campaigns must emphasize the important role of forest ecosystems in supporting both local and global environmental health. Restored and newly reforested areas could function as ecological peace corridors, aiding regional demilitarization by removing military infrastructure and establishing buffer zones that promote peace and stability.

## Conclusions

The study addressed the need for the accurate and reliable information on forest cover, dominate leaf type and the delineation of forest losses, derived from Sentinel-2 satellite data using machine learning algorithms. We demonstrated that locally trained change detection models can provide reliable information on forest cover loss, outperforming globally trained products such as the Global Forest Change (GFC) dataset. Our findings confirm that the military conflict has a significant, destructive impact on the Ukraine’s forest ecosystems. Since the beginning of the war, the total area of forest loss has doubled, and the proportion of forest affected by fire has increased considerably. Moreover, the high proportion of forest conversion to non-wood land cover may indicate a growing demand for wood following the military aggression, including increased clear cutting and illegal logging, likely driven by weakened governance and reduced enforcement of environmental regulations. This research provides essential insights into forest dynamics, contributing to a broader understanding of the environmental impacts of the ongoing conflict and additionally offering valuable support to decision-makers in managing natural resources during and after the war.

## Data Availability

The data that support the findings of this study are available from the corresponding author, upon reasonable request.

## References

[1] Author, C. C., Jonckheere, P., Nackaerts, I., Muys, K., Lambin, E. & B. & Review articledigital change detection methods in ecosystem monitoring: a review. *Int. J. Remote Sens.* **25**, 1565–1596. 10.1080/0143116031000101675 (2004).

[2] Tariq, A. et al. Modelling, mapping and monitoring of forest cover changes, using support vector machine, kernel logistic regression and Naive Bayes tree models with optical remote sensing data. *Heliyon* **9**, e13212. 10.1016/j.heliyon.2023.e13212 (2023). 36785833 10.1016/j.heliyon.2023.e13212 PMC9918775

[3] Zhao, H., Zhao, D., Jiang, X., Zhang, S. & Lin, Z. Assessment of Urban Forest Ecological Benefit Based on the i-Tree Eco Model—A Case Study of Changchun Central City. *Forests* **14**, 1304 (2023).

[4] Bennett, M. M., Van Den Hoek, J., Zhao, B. & Prishchepov, A. V. Improving satellite monitoring of armed conflicts. *Earth’s Future*. **10**, 443. 10.1029/2022ef002904 (2022).

[5] Shumilo, L. et al. Conservation policies and management in the Ukrainian Emerald network have maintained reforestation rate despite the war. *Commun. Earth Environ.* **4**10.1038/s43247-023-01099-4 (2023).

[6] Irland, L. et al. Russian invasion: rapid assessment of impact on ukraine’s forests. *Наукові праці Лісівничої академії наук України*. 10.15421/412312 (2024).

[7] Myroniuk, V. et al. Nationwide remote sensing framework for forest resource assessment in war-affected Ukraine. *For. Ecol. Manag.* **569**, 122156. 10.1016/j.foreco.2024.122156 (2024).

[8] Matsala, M., Odruzhenko, A., Sydorenko, S. & Sydorenko, S. War threatens 18% of protective plantations in Eastern agroforestry region of Ukraine. *For. Ecol. Manag.* **578**, 122361. 10.1016/j.foreco.2024.122361 (2025).

[9] Cazzolla Gatti, R., Cortès Lobos, R. B., Torresani, M. & Rocchini, D. An early warning system based on machine learning detects huge forest loss in Ukraine during the war. *Global Ecol. Conserv.* **58**, e03427. 10.1016/j.gecco.2025.e03427 (2025).

[10] Kussul, N., Yailymova, H. & Drozd, S. in *12th International Conference on Dependable Systems, Services and Technologies (DESSERT).* 1–5. (2022).

[11] Aimaiti, Y., Sanon, C., Koch, M., Baise, L. G. & Moaveni, B. War related Building damage assessment in Kyiv, Ukraine, using Sentinel-1 radar and Sentinel-2 optical images. *Remote Sens.* **14**, 6239. 10.3390/rs14246239 (2022).

[12] Holloway, J. & Mengersen, K. Statistical machine learning methods and remote sensing for sustainable development goals: A review. *Remote Sens.* **10**, 1365. 10.3390/rs10091365 (2018).

[13] Breiman, L. Random forests. *Mach. Learn.* **45**, 5–32 10.1023/a:1010933404324 (2001).

[14] Kluczek, M., Zagajewski, B. & Kycko, M. Combining multitemporal optical and radar satellite data for mapping the Tatra mountains Non-Forest plant communities. *Remote Sens.* **16**, 1451. 10.3390/rs16081451 (2024).

[15] Liu, B., Gao, L., Li, B., Marcos-Martinez, R. & Bryan, B. A. Nonparametric machine learning for mapping forest cover and exploring influential factors. *Landscape Ecol.* **35**, 1683–1699. 10.1007/s10980-020-01046-0 (2020).

[16] Belenok, V. et al. Machine learning based combinatorial analysis for land use and land cover assessment in Kyiv City (Ukraine). *J. Appl. Remote Sens.* **17**, 014506-014506. 10.1117/1.jrs.17.014506 (2023).

[17] Tokar, O., Havryliuk, S., Korol, M., Vovk, O. & Kolyasa, L. in *Advances in Intelligent Systems and Computing III.* (eds Natalia Shakhovska & Mykola O. Medykovskyy) 48–64 (Springer International Publishing, 2018).

[18] Hościło, A. & Lewandowska, A. Mapping forest type and tree species on a regional scale using Multi-Temporal Sentinel-2 data. *Remote Sens.* **11**, 929. 10.3390/rs11080929 (2019).

[19] Waśniewski, A., Hościło, A. & Zagajewski, B. & Moukétou-Tarazewicz, D. Assessment of Sentinel-2 Satellite Images and Random Forest Classifier for Rainforest Mapping in Gabon. *Forests* **11**, 941 (2020).

[20] Meng, S., Pang, Y., Huang, C. & Li, Z. Improved forest cover mapping by harmonizing multiple land cover products over China. *GIScience Remote Sens.* **59**, 1570–1597. 10.1080/15481603.2022.2124044 (2022).

[21] Pacheco-Pascagaza, A. M. et al. Near Real-Time Change Detection System Using Sentinel-2 and Machine Learning: A Test for Mexican and Colombian Forests. *Remote Sensing*14, 707 (2022).

[22] Zhu, Q., Guo, X., Li, Z. & Li, D. A review of multi-class change detection for satellite remote sensing imagery. *Geo-spatial Inform. Sci.* **27**, 1–15. 10.1080/10095020.2022.2128902 (2024).

[23] Hansen, M. C. et al. High-Resolution global maps of 21st-Century forest cover change. *Science* **342**, 850–853. 10.1126/science.1244693 (2013). 24233722 10.1126/science.1244693

[24] Rynkiewicz, A., Hościło, A., Aune-Lundberg, L., Nilsen, A. B. & Lewandowska, A. Detection and Quantification of Vegetation Losses with Sentinel-2 Images Using Bi-Temporal Analysis of Spectral Indices and Transferable Random Forest Model. *Remote Sens.* **17**, 979 (2025).

[25] Ministry of Environmental Protection and Natural Resources of Ukraine. Ecological Passports. (2022). Available at: https://mepr.gov.ua/diyalnist/napryamky/ekologichnyj-monitoryng/ekologichni-pasporty/ (accessed 2022).

[26] Zanaga, D., Van De Kerchove, R., De Keersmaecker, W., Souverijns, N., Brockmann, C., Quast, R., Wevers, J., Grosu, A., Paccini, A., Vergnaud, S., Cartus, O., Santoro, M., Fritz, S., Defourny, P. ESA WorldCover 10 m 2020 v100. European Space Agency (ESA), Zenodo (2021). https://doi.org/10.5281/zenodo.5571936

[27] Buchhorn, M., Smets, B., Bertels, L., De Roo, B., Lesiv, M., Tsendbazar, N.-E., Herold, M. & Fritz, S. Copernicus Global Land Service: Land Cover 100 m: Collection 3: Epoch 2019: Globe (Version V3.0.1) [Data set]. Zenodo (2020). https://doi.org/10.5281/zenodo.3939050

[28] Waśniewski, A., Hościło, A. & Aune-Lundberg, L. The impact of selection of reference samples and DEM on the accuracy of land cover classification based on Sentinel-2 data. *Remote Sens. Applications: Soc. Environ.* **32**, 101035. 10.1016/j.rsase.2023.101035 (2023).

[29] Gorelick, N. et al. Google Earth engine: Planetary-scale Geospatial analysis for everyone. *Remote Sens. Environ.* **202**, 18–27. 10.1016/j.rse.2017.06.031 (2017). https://doi.org/https://doi.org/

[30] Carrasco, L., O’Neil, A. W., Morton, R. D. & Rowland, C. S. Evaluating combinations of temporally aggregated Sentinel-1, Sentinel-2 and landsat 8 for land cover mapping with Google Earth engine. *Remote Sens.* **11**, 288. 10.3390/rs11030288 (2019).

[31] Hussain, M., Chen, D., Cheng, A., Wei, H. & Stanley, D. Change detection from remotely sensed images: from pixel-based to object-based approaches. *ISPRS J. Photogrammetry Remote Sens.* **80**, 91–106. 10.1016/j.isprsjprs.2013.03.006 (2013).

[32] Jin, S. et al. A comprehensive change detection method for updating the National land cover database to circa 2011. *Remote Sens. Environ.* **132**, 159–175. 10.1016/j.rse.2013.01.012 (2013). https://doi.org/https://doi.

[33] Filho, W. L. et al. The environment as the first victim: the impacts of the war on the preservation areas in Ukraine. *J. Environ. Manage.* **364**, 121399. 10.1016/j.jenvman.2024.121399 (2024). 38878570 10.1016/j.jenvman.2024.121399

[34] Ottosen, T. B., Petch, G., Hanson, M. & Skjøth, C. A. Tree cover mapping based on Sentinel-2 images demonstrate high thematic accuracy in Europe. *Int. J. Appl. Earth Obs. Geoinf.* **84**, 101947. 10.1016/j.jag.2019.101947 (2020). 35125983 10.1016/j.jag.2019.101947 PMC8804947

[35] Galiatsatos, N. et al. An assessment of global forest change datasets for National forest monitoring and reporting. *Remote Sens.* **12**, 1790. 10.3390/rs12111790 (2020).

[36] Matsala, M. et al. War drives forest fire risks and highlights the need for more ecologically-sound forest management in post-war Ukraine. *Sci. Rep.* **14**, 4131. 10.1038/s41598-024-54811-5 (2024). 10.1038/s41598-024-54811-5 PMC10876951 38374396

[37] Brockerhoff, E. G. et al. Forest biodiversity, ecosystem functioning and the provision of ecosystem services. *Biodivers. Conserv.* **26**, 3005–3035. 10.1007/s10531-017-1453-2 (2017).

[38] Mori, A. S., Lertzman, K. P. & Gustafsson, L. Biodiversity and ecosystem services in forest ecosystems: a research agenda for applied forest ecology. *J. Appl. Ecol.* **54**, 12–27. 10.1111/1365-2664.12669 (2017).

[39] Gallo-Cajiao, E. et al. Implications of russia’s invasion of Ukraine for the governance of biodiversity conservation. *Front. Conserv. Sci.* **4**, 989019. 10.3389/fcosc.2023.989019 (2023).

